# Satisfaction regarding shared decision-making for operative and non-operative hip fracture treatment in frail older adults from a proxy-perspective: a mixed methods study

**DOI:** 10.1136/bmjopen-2026-117789

**Published:** 2026-10-06

**Authors:** Paulieke C Oosterwijk, Miliaan L Zeelenberg, Henk J Schuijt, Detlef Van der Velde, Dennis Den Hartog, Taco Gosens, Pieter Joosse, Sverre A I Loggers, Thomas M P Nijdam, Johannes J M van Delden, Hugo H Wijnen, Michael H J Verhofstad, Esther M M Van Lieshout, Hanna C Willems, NOM-Implementation study group, F Sohl

**Affiliations:** 1 Department of Trauma Surgery, St Antonius Hospital, Utrecht, The Netherlands; 2 Trauma Research Unit Department of Surgery, Erasmus MC, University Medical Center Rotterdam, Rotterdam, The Netherlands; 3 Department of Internal Medicine and Geriatrics, Amsterdam University Medical Centres, Amsterdam, The Netherlands; 4 Department of Orthopedics and Trauma Surgery, Elisabeth-TweeSteden Ziekenhuis, Tilburg, The Netherlands; 5 Department of Medical and Clinical Psychology, Tilburg School of Social and Behavioral Sciences, Tilburg University, Tilburg, The Netherlands; 6 Department of Surgery, Noordwest Ziekenhuisgroep, Alkmaar, The Netherlands; 7 Department of Bioethics and Health Humanities, Julius Center for Health Sciences and Primary Care, Utrecht, The Netherlands; 8 Department of Clinical Geriatrics, Rijnstate Hospital, Arnhem, The Netherlands

**Keywords:** Patient Satisfaction, Hip Fractures, Frail Elderly, Frailty, TRAUMA MANAGEMENT, Decision Making

## Abstract

**Objectives:**

Shared decision-making (SDM) is essential in hip fracture treatment, as both operative and non-operative management are viable options in a select group of frail older adults with a very limited life expectancy. The aim of this study is to evaluate patient and proxy satisfaction with SDM and their final decision and to gain insight into their experiences.

**Design:**

A multicentre study with a concurrent mixed methods design.

**Setting:**

The study was conducted in 14 hospitals across the Netherlands.

**Participants:**

Proxies from a select group of frail older adults, with a limited life expectancy, with a hip fracture. Of the 167 patients and proxies who engaged in SDM, 1 patient and 79 proxies were interviewed.

**Interventions:**

SDM regarding operative and non-operative hip fracture treatment.

**Primary and secondary outcome measures:**

Patient-specific and treatment-specific characteristics were collected from the Dutch Hip Fracture Audit. Semistructured interviews provided quantitative and qualitative data regarding satisfaction, information and attention for patients wishes.

**Results:**

The median age was 87 years (P_25_-P_75_ 82–92), and 55 (69%) were female. In 52 patients, non-operative management was the final decision. The median satisfaction score regarding the chosen treatment was 8 out of 10 (P_25_-P_75_ 7–9). Contributing factors include acknowledging patients’ frailty and ensuring treatment consistency. Access to adequate and honest information and sufficient time allocated was crucial. In 23/78 (30%) cases, participants missed information on technical details and aftercare.

**Conclusions:**

After SDM with frail older hip fracture patients and their proxies, most participants were satisfied with the final decision. However, there are several considerations to improve SDM. This study describes practical insights and recommendations to guide healthcare providers during the SDM process.

**Trial registration number:**

NCT06079905.

**STRENGTHS AND LIMITATIONS OF THIS STUDY**

- A thematic qualitative analysis was used to identify themes relevant to improve shared decision-making (SDM).
- Only one patient could be interviewed directly; proxies were interviewed in all other cases.
- Use of Dutch Hip Fracture Audit registry data resulted in missing data for some patients.
- Variations in clinical setting may have influenced how SDM was conducted and experienced.

## Introduction

 Shared decision-making (SDM) is crucial for aligning treatment options with patient preferences, facilitating more informed and well-understood decisions that contribute to personalised care.^1 2^ Patients and proxies feel supported and empowered through effective communication in SDM.^2^ The SDM approach is especially important in hip fracture treatment in a select group of frail older adults with a limited life expectancy, as it emphasises personalised, tailored care rather than solely focusing on the clinical aspects of hip fracture treatment.^3 4^ While operative management (OM) remains the cornerstone for management of hip fractures, non-OM (NOM) can be a viable option for frail older adults when end-of-life care aligns with patient’s preferences and the primary goal is comfort.^5 6^

Nevertheless, SDM in an acute setting can be challenging due to perceived practical barriers and uncertainty about the individual prognosis.^7 8^ Previously described barriers include the time required for an SDM conversation and the inability to arrange multidisciplinary commitment.^9^ Additional challenges include patient-related barriers, where individuals may be hindered by the emergency setting and pain or side effects from opioids, which reduce their ability to take control of the situation.^10^

Previous studies in different settings have demonstrated that barriers hindering the SDM process may lead to inadequately performed SDM.^11 12^ This can result in lower patient satisfaction and a higher rate of decision regret, particularly if patients experience complications or unexpected outcomes. As NOM gains acceptance as a treatment option for this select group of frail older adults with a hip fracture, it is important to reassess and enhance the SDM process.^4 10 13^ Implementing appropriate SDM strategies may improve both patient and proxy experiences when making decisions between OM and NOM. However, the extent to which frail older adults with a hip fracture and their proxies are satisfied with the SDM process, as well as how this can be optimised, remains unclear.

The aim of this study was to assess the satisfaction of patients and their proxies with the final decision on OM versus NOM for hip fracture treatment and to gain insights into their experience regarding the SDM process. These insights will be used to provide practical recommendations to guide healthcare providers throughout the SDM process.

## Methods

### Study design

A multicentre, mixed methods study was conducted among frail older adults with a hip fracture and their proxies, from 1 January 2024 to 1 September 2024, as part of a four-phase NOM-implementation project in 14 hospitals.^14^ In a concurrent mixed methods design, both quantitative and qualitative data were collected simultaneously during a single semistructured interview that included closed and open-ended questions (figure 1). Additional data and baseline characteristics were extracted from the Dutch Hip Fracture Audit (DHFA). This is the national quality data registry of hip fracture patients, collecting data on patients, treatment and outcomes.^15^ Both quantitative and qualitative data were analysed separately. Next, quantitative and qualitative data were concurrently triangulated, meaning both were compared and integrated to enhance the completeness and validity of the results.^16^ A mixed methods approach allowed descriptive quantification alongside in-depth qualitative exploration. This study is reported according to the Good Reporting on a Mixed Methods Study (GRAMMS) Guideline.^17^

**Figure 1:**
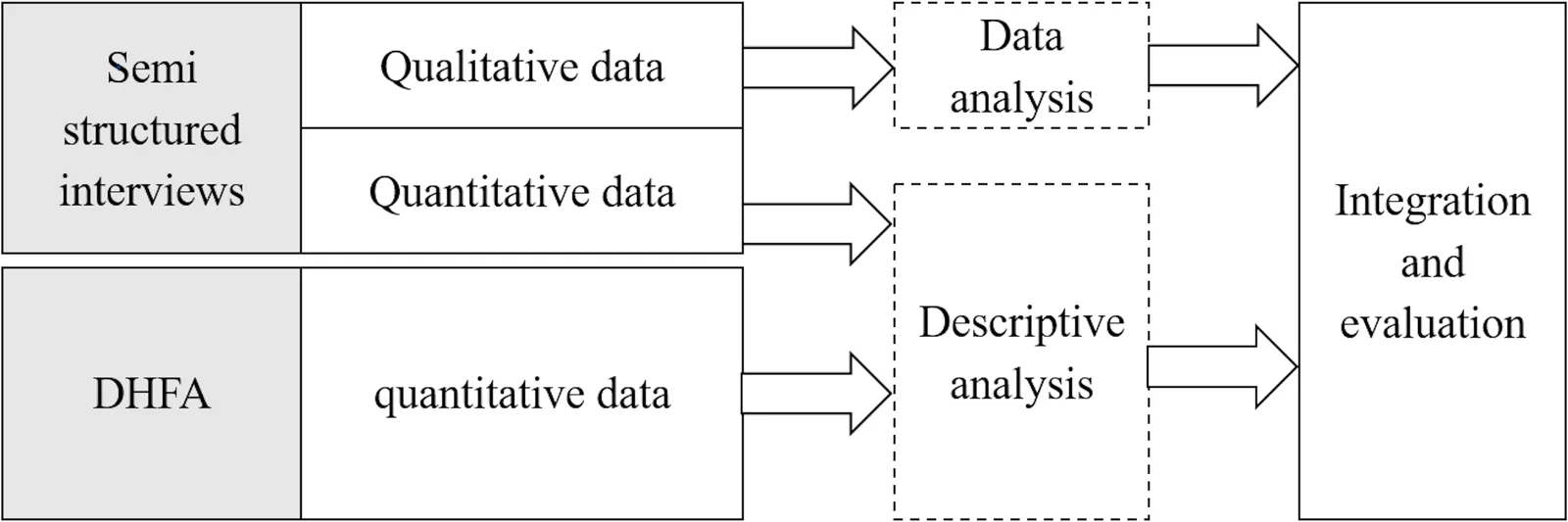
Concurrent mixed methods design. DHFA, Dutch Hip Fracture Audit.

### Context and study setting

This study was a part of the nationwide NOM-implementation project on SDM for OM and NOM of hip fractures in a selected group of frail older adults (see the Participants for further details). SDM implementation was done following an implementation protocol, which was developed in a preparation phase.^14^ During the early implementation phase, 14 hospitals in the Netherlands implemented SDM on hip fracture treatment, all level 1 or 2 trauma centres. Educational materials were developed to support the local healthcare providers during the SDM process, following the four-step SDM strategy described by Stiggelbout *et al*.^1 2^ These four steps include (1) introducing patient and proxy to their role in SDM, (2) discussing the options, (3) discussing the preferences of the patient and proxy, and (4) taking a decision together. Healthcare providers at each hospital received SDM education tailored to their needs in SDM by a certified SDM trainer (PCO or MLZ).^14^ Subsequently, the local teams performed patient selection and applied SDM on hip fracture treatment when patients met the inclusion criteria. Patient information materials were developed and distributed to the healthcare providers, who could offer them to patients and proxies when appropriate.

### Participants

Participants were patients or their proxies who attended the SDM process. Patients were eligible if they met all of the following inclusion criteria and were engaged in SDM for OM or NOM by the attending physician(s). Inclusion criteria were: patients being 70 years or older with an acute proximal femoral fracture (Arbeitsgemeinschaft für Osteosynthesefragen/Orthopaedic Trauma Association (AO/OTA) type 31), confirmed by X-ray or CT scan,^18^ who were either living in a nursing home, receiving equivalent care at home or qualifying for long-term care under the Dutch Long-Term Care Act (ie, deemed eligible for institutional long-term care).^19^ Additionally, at least one of the following frailty characteristics had to be present: either malnutrition (cachexia or body mass index <18.5 kg/m^2^), a functional ambulation category (FAC) of 2 or lower, or severe comorbidities defined as American Society of Anesthesiologists (ASA) category 4 or 5.^20 21^ Patients and/or proxies with insufficient comprehension of the Dutch language were excluded. During or after the SDM process, participants were asked if they were willing to participate in interviews and provide feedback on the SDM process and the decision. Four to six weeks after the SDM process, participants were contacted by telephone. If participants no longer wished to be interviewed, they could opt out. Participants were also excluded after at least five unsuccessful contact attempts. If a patient gave informed consent, without contact information for a proxy, but passed away, they were considered lost to follow-up. A formal sample size calculation was not made as the primary aim was implementation on a national level.^14^

### Data collection

Baseline characteristics were age, sex, living situation (institutional care facility or nursing home care at home), Katz Activities of Daily Living (ADL) score, diagnosis of dementia and ASA score.^21 22^ Furthermore, intervention characteristics of SDM were described, including what medical specialists were involved in the shared SDM process and what decision was made (OM or NOM).

Quantitative data included patient characteristics, fracture details and treatment characteristics and were partly collected from the DHFA. Other quantitative data were collected through closed questions with numeric rating scales (NRS) during the interview including satisfaction regarding the treatment decision (NRS 0–10), the valuation of the amount and content of information received (NRS 0–1 and NRS 0–5), and if there was enough attention for patient’s needs/wishes (NRS 0–1). Questions are listed in online supplemental document S2. Quantitative data were pseudonymised and then captured using Castor Electronic Data Capture (Castor EDC), a platform for data collection and management.

Qualitative data were collected through open-ended questions following the closed questions, based on the answers received. Participants were asked to explain, paraphrase and expand on their previous answers or scores, to gain a thorough understanding of experiences and perceptions of the participants on the SDM process. The interviews were recorded and stored on a secure server.

### Data analysis

Quantitative data were analysed using SPSS software V.28 (IBM, Chicago, Illinois). A descriptive analysis was performed for baseline characteristics and outcome measures in the two groups of OM and NOM. Variables were tested for normality using the Shapiro-Wilk’s test. Continuous data are reported as median with first and third quartiles and categorical data are reported as numbers with percentages. Given the baseline differences between the groups and exploratory aim of the study, no formal comparative analysis was performed, and results are presented descriptively.

Qualitative data were analysed using ATLAS.ti (V.23.1.1.0). The semistructured interviews were executed and transcribed *ad verbatim* either by PCO or MLZ. A thematic analysis following the six-step framework by Braun and Clarke^23^ was conducted.^23^ After independent familiarisation with the data, initial coding, searching and identifying key themes was done by PCO and MLZ. An intercoder agreement meeting was held until final intercoder agreement was reached.^23 24^

### Patient and public involvement statement

Patients and proxies participated through qualitative interviews but were not involved in the study design or conduct. Participants could contact the research team for additional information or feedback on the results.

## Results

### Participants

A total of 167 frail older adults and/or their proxies were engaged in SDM regarding OM or NOM for hip fracture treatment (figure 2). Following SDM, 96 participants consented to being approached for an interview. Of these, 11 participants could not be reached despite repeated attempts and five withdrew consent. In total, 80 participants were interviewed. All interviews were conducted with a proxy, except for one that was held with a patient who had surgery. Of the 79 proxies, 11 were the patient’s partners, 64 were children or children-in-law, 1 was a sibling, 1 was a cousin, and in two cases, the proxy was a befriended caregiver. In 28 (35%) patients, OM was chosen, and in 52 (65%) patients, NOM was.

**Figure 2:**
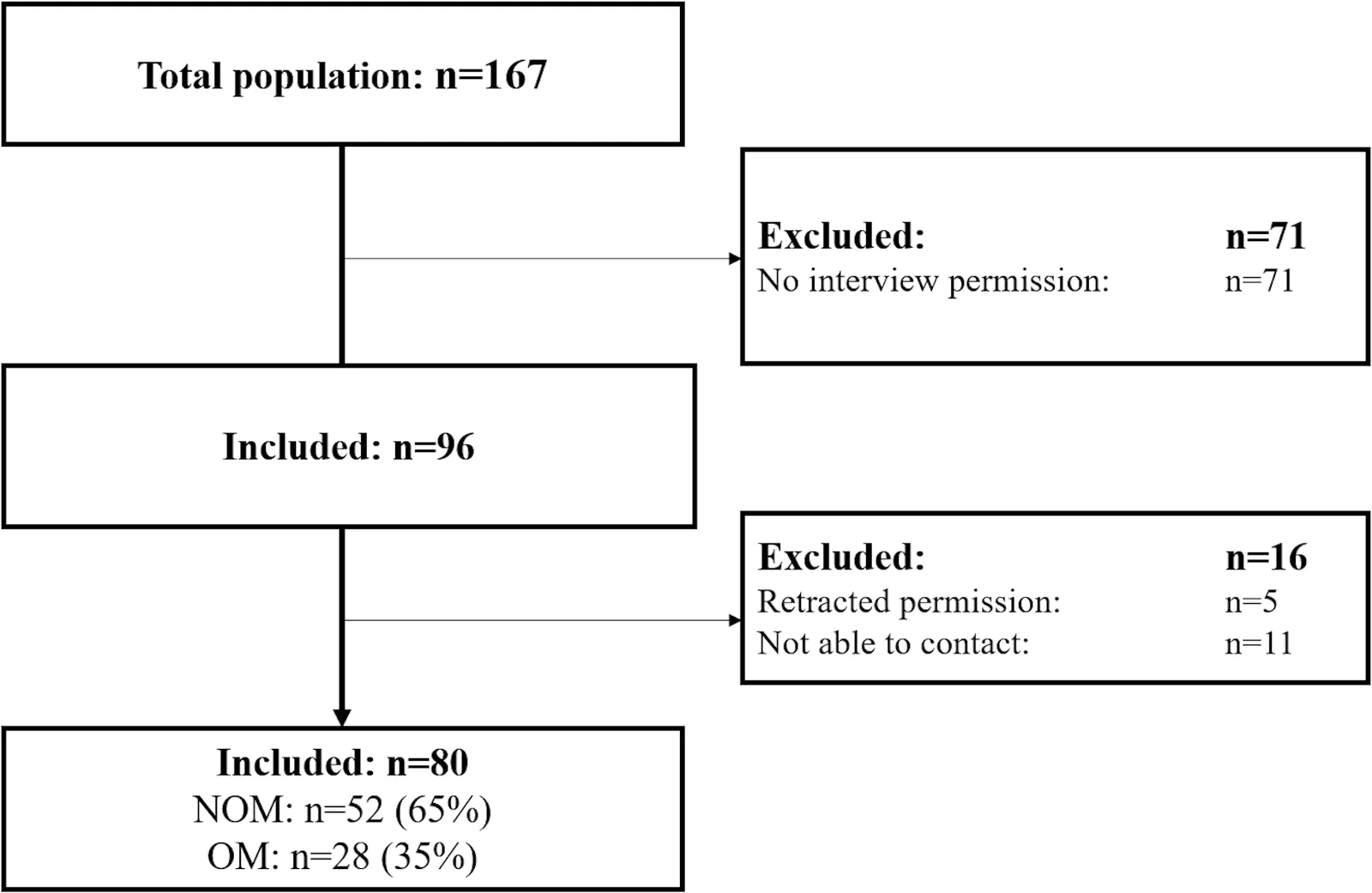
Flowchart of included patients. NOM, non-operative management; OM, operative management.

### Patient and SDM characteristics

The median age was 87 (P_25_-P_75_ 82–92) years and 55 (69%) of the patients were female (table 1). Prior to the trauma, 49 (61%) patients resided in institutional care, 22 (28%) were community-dwelling with daily ADL care. Nine (11%) patients were community-dwelling without daily ADL care but with a qualification for long-term care under the Dutch Long-Term Care Act. Pretrauma mobility was documented as follows. In the OM group, three (11%) patients were independent and four (15%) were mobile within their own home or less. In the NOM group, 9 (18%) patients were independently mobile, and 23 (47%) were mobile within their own home or less. In the total population, the median hospital length of stay was 4 days (P_25_-P_75_ 1–8). At the time of the interview, 4 to 6 weeks after the SDM process, 3 (11%) patients had died in the OM group and 40 (77%) in the NOM group. Additional baseline characteristics are described in online supplemental table S3.

**Table 1:** Baseline characteristics

	Total population (n=80)		Operative (n=28)		Non-operative (n=52)	
	n*		n*		n*	
Age (years)	80	87 (82–92)	28	85 (82–92)	52	87 (83–92)
Females	80	55 (69%)	28	17 (61%)	52	38 (73%)
Pre-trauma living status	80		28		52	
Community dwelling without daily ADL care		9 (11%)		3 (11%)		6 (12%)
Community dwelling with daily ADL care		22 (28%)		10 (36%)		12 (23%)
Institutionalised		49 (61%)		15 (54%)		34 (65%)
Pre-trauma mobility	77		28		49	
Independent		12 (16%)		3 (11%)		9 (18%)
With 1 or 2 aids		38 (49%)		21 (75%)		17 (35%)
Only within own home		20 (26%)		3 (11%)		17 (35%)
Not functional		7 (9%)		1 (4%)		6 (12%)
Dementia diagnosis	80	46 (58%)	28	13 (46%)	52	33 (64%)
HLOS (days)	80	4 (1-8)	28	8 (3-10)	52	2 (1-5)
In-hospital mortality	80	6 (8%)	28	1 (4%)	52	5 (10%)
Discharge location	74		27		47	
Own home (with care)		5 (7%)		1 (4%)		4 (9%)
Care home		49 (66%)		14 (52%)		35 (75%)
Rehabilitation institution		12 (16%)		12 (44%)		0
Hospice/other		8 (11%)		0		8 (17%)
Mortality at time of interview	80	43 (54%)	28	3 (11%)	52	40 (77%)

Data are shown as median (P_25_-P_75_) or as n (%).

n* refers to the number of patients for whom data were available.

ADL, activities of daily life; HLOS, hospital length-of-stay; MUST, Malnutrition Universal Screening Tool; SNAQ, Short Nutritional Assessment Questionnaire.

The SDM process often took place at multiple locations. SDM was at least partly conducted in the emergency department for 30/66 (46%) patients and/or at the inpatient ward for 50/66 (76%) patients (online supplemental table S4). In addition, in two patients (3%), it was partially conducted in the preoperative holding department. Data regarding the exact location of SDM and the specialties participating in SDM was missing for 14 and 3 patients, respectively.

### Satisfaction regarding the chosen treatment after SDM

During the interview, patients reported a median overall satisfaction with the chosen treatment of 8 (P_25_-P_75_ 7–9; table 2). In the total population, 59/78 (76%) of the participants described the information as sufficient, 15 (19%) participants perceived the information as being too little and four (5%) as too much. Information was sufficient in 55 (71%) participants and 73 (91%) participants felt that healthcare providers adequately considered and respected the patient’s wishes. Two proxies could not answer the questions regarding the content and volume of information as they were not present during the complete SDM process. The patient’s wishes were adequately considered in 27 (96%) participants of the OM group and in 47 (90%) participants of the NOM group.

**Table 2:** Outcomes of questionnaire on overall treatment satisfaction and SDM process

	Total population*		Operative		Non-operative	
	n†		n†		n†	
Overall satisfaction (NRS of 0–10)	80	8 (7–9)	28	9 (8–9)	52	8 (7–9)
Information amount						
Too little	78	15 (19%)	27	7 (26%)	51	8 (16%)
Sufficient		59 (76%)		19 (70%)		40 (78%)
Too much		4 (5%)		1 (4%)		3 (6%)
Missing information (yes)	78	23 (30%)		10 (37%)		13 (26%)
Enough attention for patients’ wishes (yes)	80	74 (93%)	28	27 (96%)	52	47 (90%)

n* refers to the number of patients for whom data were available.

* All interviews were conducted with a proxy, except one operative case, in which the patient was interviewed.

† Data are shown as median (P_25_–P_75_) or as n (%).

NRS, numerating rating scale; SDM, shared decision-making.

### Perceptions about the SDM process

Through thematic analysis, three themes were identified, describing underlying perceptions and contextual factors. The first two align with the quantitative questions; ‘the SDM conversation techniques’, comprising four subthemes (S1–S4) and ‘the information content and volume’ comprising three subthemes (I1–I3). Finally, an additional theme ‘logistics’ was identified, with two subthemes (L1–L2). To support the themes, quotes from the participants are provided in table 3. These themes and subthemes are presented as follows:

**Table 3:** Quotes per theme

Themes	Quotes
Satisfaction in SDM	
Balanced dialogue	‘I am, in principle, satisfied with the conversation, only at that moment, it was no longer a joint decision. So then it will come out lower than that generous 8, yes. Yes, I can honestly say, I was absolutely not dissatisfied with it. So yes, a 6 would be fine, and maybe even a bit higher. But look, as I just said, we were certainly aware that our father no longer had a strong body.’
	‘But maybe it was a tactic, or maybe it was a way to test. I don't know, of course, but that surgeon was not very positive right from the start, so to speak. The surgeon clearly had a preference. And the geriatrician too…’
Honest and open discussions	‘It came like a bolt from the blue, but they [the doctors] explained it very clearly. They didn’t beat around the bush. They simply said, this is the choice you have, and my friend, based on that information—and it was brief but very clear—just made his decision and said, I don’t want that.’
Time to talk and think	‘There was just calmness in the conversation. It was explained calmly and then again to my mother, you know. It didn’t happen in five minutes; they really took the time. I found that very pleasant.’
	‘At that moment, it just happens to you. You’re standing there, and you’re not actively thinking about it, so you accept things. You don’t really get the chance to think it through properly because, yes, the process starts immediately.’
	‘There were silences where she was thinking, and there was patience for that. I really appreciated how much patience there was to listen to her.’
Information	
Quality and completeness	‘That [the information] was exactly enough. It was very…. I also asked for very honest information. Like, don't beat around the bush, just honestly say what the advantages are for one option and what the advantages could be for the other. And, of course, also the disadvantages. And it was really, yes, very helpful in making a decision.’
Missing or incomplete information	‘That my mother could have ended up in a wheelchair or something similar, that we had talked about. But when I saw how things went, we hadn’t realistically imagined it at all. The chances were extremely small and that wasn’t entirely clear to us.’
	‘But they could have explained better what complications you can have at her age. Because I heard that if you’re at this age and have dementia, then after an operation, you might pass away within a few days.’
Supportive materials	‘That [patient information materials] could maybe be better in the future. Definitely a leaflet beforehand. Then you can calmly read what to expect and how it’s going to proceed. Yes, that kind of information.’
	‘Well, I don’t know if the things we’re discussing now would be included in such a booklet. The options, the consequences, and the risks. If that were included, it might be useful. Because we did sit around for a while or stayed with him, maybe you’ld read it during that time.’
SDM logistics	
Location matters	‘No, it really happened in the emergency room. In a separate room, so where my mother was lying, that’s where it was communicated. Yes, no, that was fine. We all know they [the doctors] are busy, so he was being called in the meantime. But yes, you know, that’s just how it is.’
	‘We spoke with the attending doctor, or the attending surgeon, in the ER. He didn’t encourage surgery, so to speak. And then later also with the geriatrician, who eventually came to the ward, so to speak, with more time and opportunity to talk. For a more in-depth conversation.’
Multidisciplinary approach	‘But if I may make a recommendation, I would say, have all the parties, all the people who are directly involved in care, be present during that conversation.’
	‘It really are the medical specialists who don’t listen to each other, don’t even look at each other. And I think there’s a lot to gain there. In healthcare in general, if people, especially medical specialists, share their knowledge and findings with each other.’

SDM, shared decision-making.

### SDM conversation techniques

#### S1: enabling SDM: facilitating an open dialogue

The way the SDM conversation was experienced played an acknowledgeable role in the satisfaction with the chosen treatment. To elaborate, participants responded positively to the information and were satisfied when the advantages and disadvantages of the options were discussed in a balanced dialogue. The availability of options was sometimes unexpected and deemed as a choice between ‘two evils’ or ‘two unfavourable scenarios’. When options were not equivalent, decisions framed as SDM were perceived as directive. This was particularly the case when the options were perceived as pressuring, one-sided, or gave the impression that the patient or their proxies' perspective was disregarded. In several cases, healthcare providers acknowledged that treatment options were not equivalent. For instance, when elevated surgical risks were identified and openly discussed, these risks often guided conversations towards choosing NOM as the final decision.

#### S2: conversation techniques: honest, open and personal discussions

Conversations perceived as blunt were perceived as such because of word choice, the impression of being dismissed and the delivery style. Although avoiding sugar-coating was important, participants considered focusing on statistics rather than the individual as harsh. Consequently, incorporating patient’s perspective was found relevant, extending beyond the advantages and disadvantages of treatment. Additionally, involving patients actively in the conversation strengthened the participants’ sense of engagement with the healthcare provider, even if the patient suffered from (partly) cognitive impairment due to dementia. Addressing the patient directly was perceived as respectful and contributed to overall satisfaction.

#### S3: providing time to talk and time to think

Participants perceived that when enough time was allocated for a calm conversation, it helped both patients and proxies feel understood and supported. The demeanour of the healthcare provider played a crucial role; standing during the conversation gave the impression that the healthcare provider was in a rush, adding pressure to make decisions quickly. The possibility to ask questions and seek clarification, both during and after the conversation, was important. The emotional intensity of the acute situation and the sudden need for decisions often made it challenging to fully and instantly comprehend the information, offering time to think and deferring decisions when possible reduced feelings of overwhelm and increased confidence in the choices made.

#### S4: involvement of proxies in SDM, before and throughout the process

Providing an opportunity in the conversations for patients or their proxies to discuss their preferences and considerations played a key role in their involvement in the conversation. It gave them the experience of being an active partner in the SDM process and they felt supported in this process. Understanding a loved one’s wishes through prior end-of-life discussions or advance care planning improved the experience of the conversation and enabled proxies to reflect on the SDM process with greater satisfaction. This was also the case when patients could openly express their preferences themselves.

### Information: content and volume

#### I1: quality of information: facilitating understanding and engagement

In general, participants were satisfied with the content and quantity of the information provided. Effective communication was characterised by clear explanations and honest discussions. The opportunity to ask questions contributed to the completeness of the information. Simple, well-formulated points, presented in a manner that avoided overwhelming the recipients, were particularly appreciated. Gradual delivery of information allowed patients and/or proxies to process the details at their own pace. When information was deemed insufficient, it was often due to the absence of key details, which hindered a comprehensive understanding of the situation and could lead to a negative perception of the SDM process in hindsight.

#### I2: incomplete communication on complications, aftercare and expectations

The most frequently missed information concerned the aftercare trajectory, which was often incomplete or unclear. Accurate details about technical options for fracture management, the likelihood of pain block effectiveness and mortality risks were considered essential. Furthermore, participants frequently noted a lack of detailed information about potential complications and their severity for both OM and NOM. They also highlighted the absence of outcome information specifically tailored to the individual patient. The absence of information was often perceived as leading to unrealistic expectations.

#### I3: the role of patient information materials in communication

Printed patient information materials were not often used. If they were used, participants underlined, it could not substitute for direct conversations, accessible explanations remained essential. Some participants expressed a desire for patient information materials, inversely a minority indicated they did not feel a need for this. However, written materials alone were not always sufficient, as some patients were unable to engage with text-based resources due to language skills or eyesight. Participants emphasised that supportive materials did help or could have helped align expectations regarding aftercare and the possibility of end-of-life outcomes.

### SDM logistics

#### L1: location matters

SDM solely in the emergency department was often described as fast-paced, disorganised, hectic or unsettling. Participants frequently perceived communication was in a directive manner when SDM took place at the emergency department. Experiences were more positive when discussions took place in a private room. At the hospital ward, participants felt there was more time and space for discussion. In addition, information was perceived as more complete, expectations were better outlined, there was more openness and time for reflection.

#### L2: the importance of a multidisciplinary approach

A multidisciplinary perspective was perceived as valuable. Conversations that included input from multiple specialties were viewed as more thorough and complete. When collaboration was inadequate, proxies perceived the SDM process as incomplete and more often stated it was lacking attention to detail. The role of geriatricians and geriatric nurses was highlighted as beneficial. Effective communication between the hospital and care facilities, both before and after discharge, was deemed essential.

### Practical implications and recommendations

The findings from the themes described above have been translated into practical implications and recommendations to guide healthcare providers with SDM for frail older adults with a hip fracture in daily practice. These recommendations are summarised and presented in figure 3 in the discussion section.

**Figure 3:**
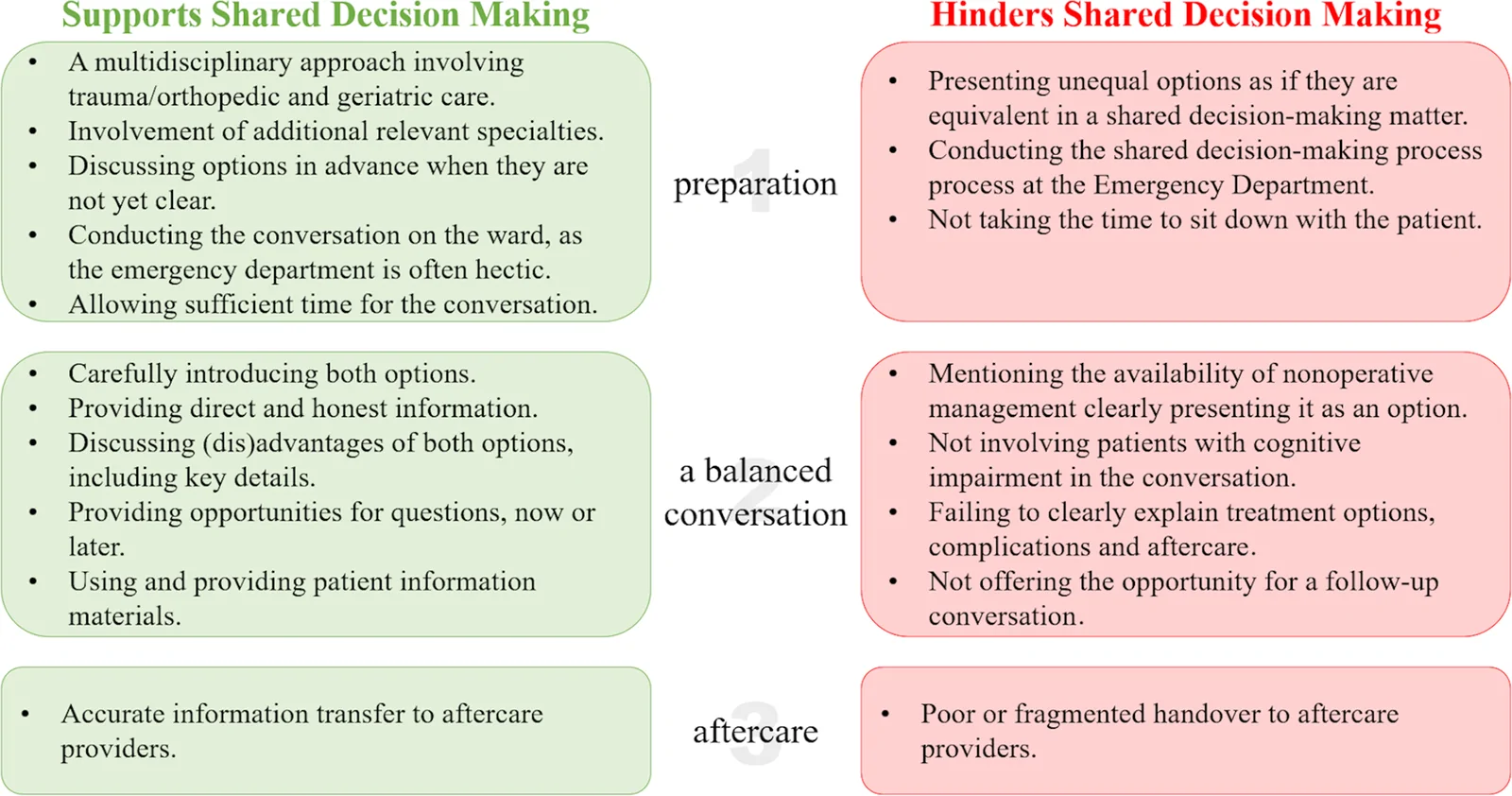
Shared decision-making in frail older adults with a hip fracture: practical insights and recommendations.

## Discussion

SDM in frail older adults with a hip fracture is increasingly recognised. Although OM remains the cornerstone in hip fracture treatment, NOM can be a viable option in a selected group of frail patients with a limited life expectancy.^5^ Implementing SDM with frail older adults selected for OM or NOM could contribute to better experiences and higher satisfaction of both patients and proxies. This study showed that after SDM most proxies were satisfied with the final decision. A balanced dialogue acknowledging patients’ frailty and ensuring consistency with their treatment were fundamental factors. Although proxies found the decisions difficult and the SDM process challenging, their involvement in this process enhanced their satisfaction. Access to adequate and honest information was fundamental to making informed choices. Key details including technical aspects of the fracture and treatment options, complications and details regarding the aftercare were often missing, although these aspects are deemed vital in this conversation. A calm, open environment with sufficient time allocated was viewed as beneficial to the decision satisfaction. Practical implications and recommendations on preparing the SDM process, conducting SDM, and providing appropriate aftercare, are described in figure 3. These are intended to guide healthcare providers during the SDM process with frail older adults with a hip fracture and their proxies.

Several previous studies have described proxies’ treatment satisfaction after OM and NOM for hip fractures in frail older adults.^5 10^ However, this is the first study investigating proxy perspectives on specifically the SDM process. Previous research in other orthopaedic/trauma patient populations has shown implementing SDM was associated with better treatment satisfaction and therapy adherence.^25^ Nevertheless, limited access to information and preparation for both healthcare providers and patients is identified as a barrier to enabling SDM in frail older adults with a hip fracture.^9^ Nationwide implementation efforts are in progress to address this issue, and the recommendations from this study could contribute to this progress and will be described in the national implementation guidelines.^14^ Despite high treatment satisfaction, not all participants in the current study were satisfied with the SDM process regarding information quantity, quality or inclusion of patient’s preferences. Providing complete, individualised information is crucial to support understanding and manage expectations, as outcome probabilities are calculated based on population data. Patient and proxies should be made aware that unexpected outcomes may occur and that predicting an individual’s prognosis is challenging. Although decision aids can support this process, they are not always used or available.^26 27^ If used, they should be readily available, for example, a hip fracture decision aid could be a valuable tool to provide guidance for the SDM process.^28^ A common challenge in SDM is achieving ‘sharedness’ in this process. It is a common misconception that patients want healthcare providers to make all difficult decisions or that informed consent equates to SDM.^29^ On the other hand, a decision is rarely truly equal. ‘Sharedness’ can be viewed as a spectrum, ranging from patient-driven decision-making to physician-driven decision-making, with true SDM in the middle.^4 30^ Depending on the possible option, the patient or the treatment goals, a different approach is necessary. SDM can then be seen as integration of two areas of expertise; the patient and/or proxy who bring insights into patient’s goals of care, and the healthcare provider who offers expertise on the treatment options.^31^ Together these perspectives contribute to deciding on care that best aligns with the patient’s preferences.

This study provides clear recommendations for improving SDM for frail older adults with a hip fracture in clinical practice. There are, however, several limitations. The perspective of only one patient could be included in this study, due to early mortality or cognitive limitations, proxies were interviewed in all other cases. While a frequent practice in research in this population, true patient-reported data could provide further valuable insights in satisfaction and decision-making among frail older patients. As numbers of included patients differed greatly between including hospitals and several participants could not be reached or declined participation. In addition, a formal non-responder analysis was not feasible, as detailed data on non-responders and the underlying patient data, for whom proxy responses were provided, were unavailable due to ethical and privacy regulations. Selection bias cannot be excluded. The authors limited the inclusion criteria to a selected group frail hip older adults, similar to the nationwide implementation study and previous research.^14 32^ However, if SDM on OM and NOM is applied to patients with less frailty, they and their proxies might experience different satisfaction rates or may face other challenges in relation to information needs and expectation management. Furthermore, although SDM followed a standardised format variations in the clinical setting, namely the high-pressure environment of the emergency department compared with a calmer ward, may have influenced the way SDM was conducted. These differences could have impacted the experiences of proxies, highlighting an important implication for daily practice. Moreover, individual experiences or the clinical course following a decision may have shaped both treatment satisfaction and the perception of the SDM process. Short-term mortality is high in this frail population, with 77% deceased after NOM at the time of the interview. Alternative approaches (eg, delayed or repeated interviews) were not considered feasible due to potential recall bias and emotional increased burden for participants. Consequently, many proxy responses were obtained in a context of bereavement, potentially introducing information bias. Qualitative responses may also have been influenced by participants’ prior quantitative responses, as interviews encouraged reflection and elaboration on earlier answers. Aligned with the exploratory aim of this study, the relatively small sample size precluded the application of methods such as propensity score matching to improve comparability between the OM and NOM groups. As such, these clinically distinct populations are not directly comparable, and findings should be interpreted as descriptive. Data for some patients were not collected or missing due to incomplete data in the DHFA. Although the proportion of missing data was limited, this may have introduced bias affecting the representativeness of the study population and the robustness of the findings. This should be considered when interpreting the findings. Finally, the personal experiences and professional background of the involved researchers may have introduced bias throughout (qualitative) data collection, analysis and interpretation.

A substantial proportion of (Dutch) surgeons have shown gaps in their understanding of SDM and find it challenging to accomplish or implement without guidance.^26 33 34^ Training healthcare providers in SDM and using a dedicated protocol for implementation, like the one related to this study, may help to improve these points.^14^ In this SDM trained context, proxies were satisfied with the SDM process and final decision but expressed several aspects that need improvement. The insights from this study provide practical implications, those will be used to enhance clinical implementation of SDM for frail older adults with a hip fracture.

## Conclusion

This study showed that, after SDM with frail older adults, proxies were generally satisfied with the final decision on hip fracture treatment. Several important factors contributing to satisfaction in the SDM process were a balanced dialogue, enough allocated time, a multidisciplinary approach and adequate sharedness of the decision. Healthcare providers should provide patients with neutral but realistic information on technical aspects, complications and aftercare.

## Supplementary material

10.1136/bmjopen-2026-117789 online supplemental file 1

## Data Availability

Data are available upon reasonable request.
