# Supplementary material for: Satisfaction regarding shared decision-making for operative and non-operative hip fracture treatment in frail older adults from a proxy-perspective: a mixed methods study
Source: BMJ Open. 2026 Oct 6;16(10):e117789. doi: 10.1136/bmjopen-2026-117789 (PMC13646964; doi:10.1136/bmjopen-2026-117789)
Supplement: Supplementary file 1 [file bmjopen-2026-117789.reviewer_comments.pdf]

PEER REVIEW HISTORY

BMJ Open publishes all reviews undertaken for accepted manuscripts. Reviewers are asked to complete a checklist review form and are provided with free text boxes to elaborate on their assessment. These free text comments are reproduced below.

ARTICLE DETAILS

Title (Provisional)

Satisfaction regarding Shared Decision Making for operative and nonoperative hip fracture treatment in frail older adults: A mixed methods study

Authors

Oosterwijk, Paulieke C.; Zeelenberg, Miliaan L.; Schuijt, Henk J.; Van der Velde, Detlef; Den Hartog, Dennis; Gosens, Taco; Joosse, Pieter; Loggers, Sverre A.I.; Nijdam, Thomas M.P.; van Delden, Johannes; Wijnen, Hugo H.; Verhofstad, Michael H.J.; Van Lieshout, Esther M.M.; Willems, Hanna C.; The NOM-Implementation study group, ,

---

VERSION 1 - REVIEW

Reviewer 1

Name Guo, Junfei

Affiliation Xi'an Jiaotong University, Department of Orthopaedic, Joint Surgery Surgery

Date 06-May-2026

COI None

This manuscript addresses a clinically relevant and timely topic — patient and proxy satisfaction with shared decision-making (SDM) in the context of operative versus nonoperative hip fracture management in frail older adults. The study fills a gap in the literature by capturing proxy perspectives on the SDM process itself, rather than solely on treatment outcomes. The mixed methods design is appropriate for the research question. However, despite its merits, the manuscript suffers from several methodological, analytical, and reporting deficiencies that substantially weaken its conclusions.

Major Concerns

1. The study purports to evaluate "patient and proxy satisfaction," yet only one patient was interviewed directly (and that patient was in the operative group). The entire dataset is therefore proxy-reported. This is acknowledged but insufficiently addressed. The title and abstract should be revised to accurately reflect that this is almost exclusively a proxy perception study. The current framing risks misleading readers.

2. Of 172 patients engaged in SDM, only 80 proxies (47%) were interviewed. Among the 92 excluded, 71 did not grant interview permission. The authors provide no analysis of whether responders differed systematically from non-responders in terms of treatment choice, patient survival, or baseline frailty. Given that 77% of NOM patients had died by the time of interview, the proxy interviews in the NOM group are heavily shaped by bereavement, which likely introduces a distinct cognitive and emotional bias (e.g., post-hoc rationalization or grief-related dissatisfaction). This is not adequately discussed.

3. The satisfaction and information adequacy measures are single-item, non-validated scales (NRS 0–10, binary yes/no). No established SDM measurement tool (e.g., SDM-Q-9, CollaboRATE, OPTION scale) was employed. This is a critical weakness for a study whose primary aim is to evaluate SDM satisfaction.

4. The authors compare satisfaction scores between the OM and NOM groups, but the groups are fundamentally non-comparable: they differ in mortality (11% vs. 77%), complication rates (39% vs. 6%), hospital length of stay, and pre-trauma mobility. The OM and NOM groups represent clinically distinct populations with vastly different trajectories. Comparing satisfaction scores between them without any adjustment or propensity matching is statistically naive and clinically misleading. The absence of significant differences likely reflects insufficient power rather than true equivalence.

5. Interviews were conducted 4–6 weeks post-SDM. By this point, 54% of patients had died. Proxy recall of the SDM conversation is inevitably colored by the patient's subsequent clinical course and death. A proxy whose loved one survived surgery may recall the conversation very differently from one whose loved one died within days of choosing NOM. This confound is not addressed in the analysis and is barely mentioned in the limitations.

Minor Concerns

1. The flowchart (Figure 2) contains an inconsistency. The text states 172 patients were engaged in SDM, but the flowchart shows n=167 as the total population. This discrepancy must be explained.

2. Table 1 reporting. The p-value for pre-trauma mobility (p=0.007) is described as a "significant difference," but the direction of the difference is counterintuitive — more NOM patients (18%) were independently mobile than OM patients (11%).

3.Data on the location of SDM and participating specialties were missing for 14 and 3 patients, respectively. ASA scores were available for only 36/80 patients. The extent of missingness and its potential impact on findings should be discussed more transparently.

4. Several grammatical errors persist (e.g., "55 (69%) was female" should be "were female"; "approval data" should be "approval date").

**Reviewer**	**2**
**Name**	**Launonen, Antti**

**Affiliation**	**Tampere University Hospital**
**Date**	**28-May-2026**
**COI**	**None**

Thank you for this needed and important publication. This is a very relevant study, which is needed more. The SDM and frailty are at the centre of our treatments in orthopaedics and traumatology at the moment and in the upcoming decades, but unfortunately there is a knowledge gap in both of them. Therefore, I am glad the authors have made an effort to narrow the gap.

Satisfaction regarding Shared Decision Making for operative and nonoperative hip fracture treatment in frail older adults: A mixed methods study

Thank you for this needed and important publication. This is a very relevant study, which is needed more. The SDM and frailty are at the centre of our treatments in orthopaedics and traumatology at the moment and in the upcoming decades, but unfortunately there is a knowledge gap in both of them. Therefore, I am glad the authors have made an effort to narrow the gap.

I have no major revisions requested. Only more clarifying ones and suggestions for readability, since there were e.g. quite a lot of national context, which is not known outside the Netherlands

The study is based on frailty and SDM. However, there is no introduction for one or the other. I am sure most readers have misconceptions about SDM, so it would be helpful to outline some basic steps. Frailty is a very abstract concept without really good metrics or definitions. That also might be a bit culturally bound, so some definition of what you have regarded as a frail patient would also be good.

126 What different domains does DHFA include? That would add a layer of understanding of the registry.

128 Triangulation; maybe a small definition. It wasn't easy to find from the referred publication.

134 Some words of national implementation – initiative, driver, etc.

154 What does the qualification mean, i.e., how do they qualify?

163 Did all participants give written informed consent, or was it from the proxy?

167 No FAC or frailty scale?

179 What is Castor?

210 You could, if you think it's relevant, write open e.g. mortality (and delirium) rate(s) to the text, as the difference between the groups seems to be high.

221 Fig 1; From 167, 87 were excluded, leading to 85 included. The 5 participants who retracted permission are essentially exclusions. At the current state, Fig 1 is not self-explanatory.

283-285 "To elaborate…" I think this is very important and worth considering to add in the conclusion.

290 How was the elevated risk of mortality received, and did it have an effect on the choice?

For me, the SDM techniques and delivering the information sections are the core of this publication. If it's not too much trouble for the authors, I feel it would help readers understand and serve as a quick reference in the future to include a figure to visualise the content. Table 4 serves a similar, base purpose, but is not visual and not so easy to memorise. A figure could be two-sided: "this thing helps" (on the other end), but "this thing is not affordable" (on the opposite end). This would be a good addition for Table 4.

VERSION 1 - AUTHOR RESPONSE

Reviewer 1:

Major Concerns

Comment 1:
The study purports to evaluate "patient and proxy satisfaction," yet only one patient was interviewed directly (and that patient was in the operative group). The entire dataset is therefore proxy-reported. This is acknowledged but insufficiently addressed. The title and abstract should be revised to accurately reflect that this is almost exclusively a proxy perception study. The current framing risks misleading readers.

Answer:
Thank you for this relevant point. We have revised the title, abstract and conclusion to more clearly reflect that the study primarily represents proxy perspectives, with input from a single patient (title on l.2, in abstract l.52-54, l.488)

Comment 2:
Of 172 patients engaged in SDM, only 80 proxies (47%) were interviewed. Among the 92 excluded, 71 did not grant interview permission. The authors provide no analysis of whether responders differed systematically from non-responders in terms of treatment choice, patient survival, or baseline frailty. Given that 77% of NOM patients had died by the time of interview, the proxy interviews in the NOM group are heavily shaped by bereavement, which likely introduces a distinct cognitive and emotional bias (e.g., post-hoc rationalization or grief-related dissatisfaction). This is not adequately discussed.

Answer:
The authors thank the reviewer for this important point. Due to ethical and privacy regulations, we did not have access to detailed information on non-responders, nor to the underlying patient data

for whom the proxies would have provided responses. As a result, a formal non-responder analysis was not feasible. We agree that this may introduce selection bias, which has now been more explicitly addressed in the Discussion section (l.449-452). In addition, the high mortality rate in the NOM group (77% at the time of interview) may have influenced the proxy perspectives, as bereavement could affect responses. We have now added this potential source of bias more clearly in the discussion (l.461-466).

Comment 3:
The satisfaction and information adequacy measures are single-item, non-validated scales (NRS 0–10, binary yes/no). No established SDM measurement tool (e.g., SDM-Q-9, CollaboRATE, OPTION scale) was employed. This is a critical weakness for a study whose primary aim is to evaluate SDM satisfaction.

Answer:
Thank you for this valuable remark. In the context of evaluating the implementation of SDM in clinical practice, a simple NRS was chosen to provide a pragmatic and feasible measure of overall satisfaction across a frail population and their proxies. Importantly, the depth and context of experiences with SDM were explored through the qualitative interviews, which allowed for a more nuanced understanding of satisfaction and decision-making processes beyond quantitative scoring. The authors agree that the inclusion of a validated SDM instrument such as the SDM-Q-9 would have strengthened the quantitative assessment and would be a valuable addition for future research.

Comment 4:
The authors compare satisfaction scores between the OM and NOM groups, but the groups are fundamentally non-comparable: they differ in mortality (11% vs. 77%), complication rates (39% vs. 6%), hospital length of stay, and pre-trauma mobility. The OM and NOM groups represent clinically distinct populations with vastly different trajectories. Comparing satisfaction scores between them without any adjustment or propensity matching is statistically naive and clinically misleading. The absence of significant differences likely reflects insufficient power rather than true equivalence.

Answer:
The authors thank the reviewer for this important and thoughtful comment. The OM and NOM groups differ substantially in terms of mortality, complications, and baseline characteristics. This is consistent with previous literature in frail hip fracture populations [2-5],. Both groups reflect a population with a very limited life expectancy. The higher mortality and earlier death in the NOM group is inherent to the clinical decision-making process, as NOM is more often chosen for the most frail patients. The aim of this study was not to compare OM and NOM as equivalent groups, nor to establish causal effects, but rather to explore experiences with shared decision-making in real-world clinical practice. In line with this aim, the presentation of the results has been revised to avoid comparative interpretation and to emphasize a descriptive approach (l.245-251, 275, fig 1, table 1 and table 2). Accordingly, baseline characteristics and satisfaction scores should be interpreted as descriptive and exploratory.

While methods such as propensity score matching could theoretically improve comparability, the relatively small sample size would have substantially reduced the study population and limited insights into this clinically relevant subgroup. this is now acknowledged as a limitation in the discussion section (l.468-472). In addition, strict matching may reduce real-world representativeness and obscure clinically meaningful differences. So, we have chosen to represent the two groups as they were included in our study, but now adjusted the text and tables to prevent comparisons between the groups.

Comment 5:
Interviews were conducted 4–6 weeks post-SDM. By this point, 54% of patients had died. Proxy recall of the SDM conversation is inevitably colored by the patient's subsequent clinical course and death. A proxy whose loved one survived surgery may recall the conversation very differently from one whose loved one died within days of choosing NOM. This confound is not addressed in the analysis and is barely mentioned in the limitations.

Answer:
The authors agree that proxy recall of the SDM conversation may have been influenced by the patient's subsequent clinical course, including survival or death. Given the high short-term mortality in this frail population (with 77% deceased after NOM at the time of interview), many responses were obtained in a context of bereavement. This is now more explicitly addressed this in the limitations, acknowledging that this may have introduced information bias (l.461-465).

Minor Concerns

Comment 1:
The flowchart (Figure 2) contains an inconsistency. The text states 172 patients were engaged in SDM, but the flowchart shows n=167 as the total population. This discrepancy must be explained.

Answer:
Thank you for identifying this inconsistency. The flowchart (Figure 2) correctly reflects the total population (n=167), and the text has now been revised to ensure consistency (l.228).

Comment 2:
Table 1 reporting. The p-value for pre-trauma mobility (p=0.007) is described as a "significant difference," but the direction of the difference is counterintuitive — more NOM patients (18%) were independently mobile than OM patients (11%).

Answer:
The authors thank the reviewer for this observation. This point has been addressed through revision of the manuscript, in line with our response to Comment 4. The results are now presented descriptively, and the reference to a statistically significant difference has been removed to avoid potential misinterpretation and comparisons.

Comment 3:
Data on the location of SDM and participating specialties were missing for 14 and 3 patients, respectively. ASA scores were available for only 36/80 patients. The extent of missingness and its potential impact on findings should be discussed more transparently.

Answer:
Data for some characteristics were missing due to incomplete registration in the Dutch Hip Fracture Audit. Although the proportion of missing data was limited for most outcomes, missingness may not have been random and could have introduced bias. This may have affected the representativeness of the study population and the robustness of the findings. This limitation has now been more clearly addressed in the manuscript (l. 472-475).

Comment 4:
Several grammatical errors persist (e.g., "55 (69%) was female" should be "were female"; "approval data" should be "approval date").

Answer:
The authors thank the reviewer for carefully noting these grammatical errors. These have been corrected throughout the manuscript.

Reviewer 2
I have no major revisions requested. Only more clarifying ones and suggestions for readability, since there were e.g. quite a lot of national context, which is not known outside the Netherlands.

Comment 1:
The study is based on frailty and SDM. However, there is no introduction for one or the other. I am sure most readers have misconceptions about SDM, so it would be helpful to outline some basic steps. Frailty is a very abstract concept without really good metrics or definitions. That also might be a bit culturally bound, so some definition of what you have regarded as a frail patient would also be good.

Answer:
The authors thank the reviewer for this clarification. Indeed, shared decision-making encompasses various strategies; in this study, we implemented the framework developed by Stiggelbout et al [6]. This approach has been described in more detail in our previously published study protocol [1]. The four steps of the SDM process are now more clearly described in the Methods section (l.145-147)
Frailty is a complex and multifaceted concept that can be described in extensive detail. To maintain focus and clarity, we have chosen to explicitly describe the frailty characteristics used for inclusion within the inclusion criteria, consistent with our prepublished protocol.

Comment 2:
126 What different domains does DHFA include? That would add a layer of understanding of the registry.

Answer:
The DHFA contains data on patient characteristics, treatment, and outcomes; this has been more clearly described in the Methods section (l.127-128).

Comment 3:
128 Triangulation; maybe a small definition. It wasn't easy to find from the referred publication.

Answer:
A definition was given for the term triangulation (l.130).

Comment 4:
134 Some words of national implementation – initiative, driver, etc.

Answer:
Thank you for this comment. As the manuscript already provides a summary of the context and setting, readers are referred to the prepublished protocol for more comprehensive details on the national implementation.

Comment 5
154 What does the qualification mean, i.e., how do they qualify?

Answer:
This has been clarified in the revised manuscript (l.160-161).

Comment 6:
163 Did all participants give written informed consent, or was it from the proxy?

Answer:
Informed consent was obtained from patients, or from their proxy when patients were unable to provide consent themselves. This has been clarified in the Methods section (l. 223-224)

Comment 7:
167 No FAC or frailty scale?

Answer:
Frailty was operationalized using characteristics available in the DHFA. Although no formal frailty score was included in the registry, functional status was partly captured using the Katz ADL score. The inclusion of a validated frailty measure, such as clinical frailty scale, would have further strengthened the assessment, but such data were not available

Comment 8:
179 What is Castor?

Answer:
We have clarified in the manuscript that Castor Electronic Data Capture (Castor EDC) is a platform for data collection and management (l.188).

Comment 9:
210 You could, if you think it's relevant, write open e.g. mortality (and delirium) rate(s) to the text, as the difference between the groups seems to be high.

Answer:
We thank the reviewer for this suggestion. The mortality rates and their differences between groups are described later in the manuscript (Results section, l.249-251). Delirium was not included, as it was not captured in the available dataset. The discussion has been revised to further elaborate on the high short-term mortality. (l.462-464)

Comment 10:
221 Fig 1; From 167, 87 were excluded, leading to 85 included. The 5 participants who retracted permission are essentially exclusions. At the current state, Fig 1 is not self-explanatory.

Answer:
The Flowchart in Fig 2 is revised to improve clarity.

Comment 11:
283-285 "To elaborate…" I think this is very important and worth considering to add in the conclusion.

Answer:
The role of a balanced dialogue was more explicitly added to the conclusion (l.397)

Comment 12:
290 How was the elevated risk of mortality received, and did it have an effect on the choice?

Answer:
We thank the reviewer for this important question. The elevated short-term mortality risk is an essential aspect of the SDM context in this frail population. As described in the revised manuscript, short-term mortality was high, with 77% of patients in the NOM group deceased at the time of the

interview 4-6 weeks after suffering hip fracture. As a result, many responses were obtained from proxies in a context of bereavement, which may have influenced both the perception of the decision-making process and reported satisfaction. While our qualitative data did not systematically capture how the explicitly communicated mortality risk affected the initial treatment choice, both the anticipated prognosis and subsequent clinical course are likely to have shaped participants' reflections on the decision. This has now been further elaborated in the manuscript (l.481-486)

Comment 13:
For me, the SDM techniques and delivering the information sections are the core of this publication. If it's not too much trouble for the authors, I feel it would help readers understand and serve as a quick reference in the future to include a figure to visualise the content. Table 4 serves a similar, base purpose, but is not visual and not so easy to memorise. A figure could be two-sided: "this thing helps" (on the other end), but "this thing is not affordable" (on the opposite end). This would be a good addition for Table 4

Answer:
Thank you for this valuable suggestion. We have added a figure (figure 3) reflecting the content of Table 4 to enhance its visual presentation, which could replace Table 4 in the manuscript. Please let us know if this more visual and colorful figure satisfies and can replace table 4.

References:

1. *Zeelenberg ML, Oosterwijk PC, Willems HC, Gosens T, Den Hartog D, Joosse P, et al. Shared decision-making for non-operative management versus operative management of hip fractures in selected frail older adults with a limited life expectancy: a protocol for a nationwide implementation study. BMJ Open 2024;14:e083429. https://doi.org/10.1136/BMJOPEN-2023-083429.*
2. Loggers SAI, Willems HC, Van Balen R, Gosens T, Polinder S, Ponsen KJ, et al*. Evaluation of Quality of Life After Nonoperative or Operative Management of Proximal Femoral Fractures in Frail Institutionalized Patients: The FRAIL-HIP Study*. JAMA Surg 2022;157:424–34. doi:
3. Schuijt HJ, Lehmann LS, Javedan H, et al*. A culture change in geriatric traumatology: holistic and patient-tailored care for frail patients with fractures. J* Bone Joint Surg Am 2021;103:e72. doi: 10.2106/JBJS.20.02149
4. Sullivan NM, Blake LE, George M, Mears SC. *Palliative Care in the Hip Fracture Patient.* Geriatr Orthop Surg Rehabil 2019;10. Doi: 10.1177/2151459319849801.
5. Murray IR, Biant LC, Clement NC, Murray SA. *Should a hip fracture in a frail older person be a trigger for assessment of palliative care needs?* BMJ Support Palliat Care 2011;1:3–4. doi: 0.1136/BMJSPCARE-2011-000027.
6. Stiggelbout AM, Pieterse AH, De Haes JCJM. *Shared decision making: Concepts, evidence, and practice.* Patient Educ Couns 2015;98:1172–9. Doi: 10.1016/j.pec.2015.06.022.

VERSION 2 - REVIEW

**Reviewer**	**2**
**Name**	**Launonen, Antti**

Affiliation Tampere University Hospital

Date 09-Aug-2026

COI

Thank you for Figure 3. That's something that fits my mindset well. I will leave it to the authors' discretion whether it should replace Table 4 or not.
