# Supplementary material for: Satisfaction regarding shared decision-making for operative and non-operative hip fracture treatment in frail older adults from a proxy-perspective: a mixed methods study
Source: BMJ Open. 2026 Oct 6;16(10):e117789. doi: 10.1136/bmjopen-2026-117789 (PMC13646964; doi:10.1136/bmjopen-2026-117789)
Supplement: Supplementary file 2 [file bmjopen-16-10-s001.docx]

**Supplementary File 1**

**Supplemental Table S1** Contributors in NOM-Implementation study group

| **Institution** | **Location** | **First name** | **Last name** | **Title/Academic degree** | **Medical specialty** |
| --- | --- | --- | --- | --- | --- |
| Alrijne ziekenhuis | Leiderdorp | E. | Sohl | MD PhD | Geriatrics |
| Alrijne ziekenhuis | Leiderdorp | J.A. | Jansen | MD | Orthopedics |
| Alrijne ziekenhuis | Leiderdorp | M. | Leijnen | MD PhD | Surgery |
| Canisius Wilhelmina ziekenhuis | Nijmegen | W.A.H. | Van der Stappen | MD | Surgery |
| Canisius Wilhelmina ziekenhuis | Nijmegen | J. | Jansen | MD PhD | Orthopedics |
| Canisius Wilhelmina ziekenhuis | Nijmegen | M.E. | Peters-Kop | MANP | Geriatric trauma unit |
| Catharina ziekenhuis | Eindhoven | A.H. | Van der Veen | MD PhD | Surgery |
| Catharina ziekenhuis | Eindhoven | N.C. | Schepel | MD | Orthopedics |
| Catharina ziekenhuis | Eindhoven | J.A.M. | Wilmer | MD | Geriatrics |
| Dijklander ziekenhuis | Hoorn | J. | Steens | MD PhD | Orthopedics |
| Dijklander ziekenhuis | Hoorn | J. | Winkelhagen | MD | Surgery |
| Dijklander ziekenhuis | Hoorn | N.M.F. | Noorda | MD | Geriatrics |
| Elisabeth TweeSteden ziekenhuis | Tilburg | H.A.A.M. | Maas | MD PhD | Geriatrics |
| Franciscus ziekenhuis | Rotterdam | O. | Wijers | MD PhD | Surgery |
| Franciscus ziekenhuis | Rotterdam | V. | Vis | MPA | Geriatric trauma unit |
| Maasstad ziekenhuis | Rotterdam | D. | Van der Stap | MD | Geriatrics |
| Maasstad ziekenhuis | Rotterdam | G.R. | Roukema | MD PhD | Surgery |
| Martini ziekenhuis | Groningen | E. | Bosma | MD | Surgery |
| Martini ziekenhuis | Groningen | T.M. | Van Raaij | MD PhD | Orthopedics |
| Martini ziekenhuis | Groningen | A.M. | Van der Knaap | MD | Geriatrics |
| Medisch spectrum Twente | Enschede | R. | de Groot | MD PhD | Surgery |
| Medisch spectrum Twente | Enschede | A.V.C.M. | Zeegers | MD PhD | Orthopedics |
| OLVG | Amsterdam | H.A. | Formijne Jonkers | MD PhD | Surgery |
| OLVG | Amsterdam | D.H.R. | Kempen | MD PhD | Orthopedics |
| OLVG | Amsterdam | K. | De Vries | MD | Geriatrics |
| Rijnstate ziekenhuis | Arnhem | A.F. | Pull ter Gunne | MD PhD | Surgery |
| Rijnstate ziekenhuis | Arnhem | M.P. | Somford | MD PhD | Orthopedics |
| Rode Kruis ziekenhuis | Beverwijk | E. | Tanis | MD PhD | Surgery |
| Rode Kruis ziekenhuis | Beverwijk | J.H. | Duits | MD | Geriatrics |
| Rode Kruis ziekenhuis | Beverwijk | R.A. | Zandbergen | MD | Orthopedics |
| St. Antonius ziekenhuis | Leidse Rijn | N.C. | Leegwater | MD PhD | Orthopedics |
| St. Antonius ziekenhuis | Leidse Rijn | O.C. | Geraghty | MD PhD | Geriatrics |
| Vilans | Utrecht | R.E. | Pel-Littel | PhD | Health care research |
| Ziekenhuisgroep Twente | Almelo | J.H. | Hegeman | MD PhD | Surgery |
| Ziekenhuisgroep Twente | Almelo | E.M. | Regtuijt | MD | Geriatrics |

MD, Doctor of medicine; MANP, Master of Advanced Nursing Practice; MPA, Master Physician Assistant; PhD, Doctor of philosophy.

**Supplemental document S1** Semi-structured interview questionnaire
*After you/your proxy broke your/their hip, the decision on whether or not you would undergo surgery was made in close consultation with the surgeon and geriatric specialist. To gain insight into whether patients and/or their relatives are satisfied with the decision, we would like to ask you to answer the following question:*

- *If you/your proxy underwent surgery: How would you rate your satisfaction 0 – 10 in regard to the decision to operate, that was made following SDM with your health care provider?*
- *If you/your proxy did not underwent surgery: How would you rate your satisfaction 0 – 10 in regard to the decision to not operate, that was made following SDM with your health care provider*

|  |  | Utterly  dissatisfied | | | | | Utterly  satisfied | | | | | |
| --- | --- | --- | --- | --- | --- | --- | --- | --- | --- | --- | --- | --- |
|  |  | 0 | 1 | 2 | 3 | 4 | 5 | 6 | 7 | 8 | 9 | 10 |
| 1. | Satisfaction: | ☐ | ☐ | ☐ | ☐ | ☐ | ☐ | ☐ | ☐ | ☐ | ☐ | ☐ |

*Your healthcare practitioners have given you information regarding the advantages and disadvantages regarding operative management and nonoperative management. We would like to know what you thought of this information.*

| 2. | What did you think of the amount of information? | ☐ Way too much  ☐ A little too much  ☐ Exactly enough  ☐ A little too little  ☐ Way too little |
| --- | --- | --- |
| 3. | Were you satisfied with the content of the information? | ☐ Yes, everything i wanted to know was discussed.  ☐ No, i would have wanted to know more about: …… |
| 4. | Do you think enough attention was given to what was important for you/your proxy when choosing the treatment? | ☐ Yes, the doctors have choosen the best fitting treatment for me/my proxy.  ☐ No, there was not enough attention for wat was important for me/my proxy |
|  | If not, could you elaborate: |  |

**Supplemental Table S3** Additional patient characteristics, including fracture type, operation characteristics and in-hospital complications

|  | **Total population** | | **Operative** | | **Nonoperative** | |
| --- | --- | --- | --- | --- | --- | --- |
|  | n* |  | n* |  | n* |  |
| **Fracture type** | 74 |  | 28 |  | 46 |  |
| Femoral neck |  | 46 (62%) |  | 15 (54%) |  | 31 (67%) |
| Trochanteric |  | 27 (37%) |  | 12 (43%) |  | 15 (33%) |
| Subtrochanteric |  | 1 (1%) |  | 1 (4%) |  | 0 |
| **ASA score** | 36 |  | 27 |  | 9 |  |
| 2 |  | 3 (8%) |  | 2 (7%) |  | 1 (11%) |
| 3 |  | 18 (50%) |  | 15 (55%) |  | 3 (33%) |
| 4 |  | 15 (42%) |  | 10 (37%) |  | 5 (56%) |
| **Operative fixation** | 28 |  | 28 |  | NA |  |
| Hemiarthroplasty |  | 15 (54%) |  | 15 (54%) |  | NA |
| Intramedullary nail |  | 13 (46%) |  | 13 (46%) |  | NA |
| **In-hospital complications** | 80 |  | 28 |  | 52 |  |
| Any |  | 14 (18%) |  | 11 (39%) |  | 3 (6%) |
| Anemia |  | 3 |  | 3 |  | 0 |
| Delirium |  | 10 |  | 7 |  | 3 |
| Fall |  | 1 |  | 1 |  | 0 |
| Heart failure |  | 1 |  | 1 |  | 0 |
| Kidney failure |  | 1 |  | 1 |  | 0 |
| Pneumonia |  | 1 |  | 1 |  | 0 |
| Pressure sores |  | 1 |  | 1 |  | 0 |
| UTI |  | 2 |  | 1 |  | 1 |

**^*^**Data are shown as median (P_25_-P_75_) or as n (%). n* refers to the number of patients for whom data were available.

**Supplemental Table S4** Location(s) of SDM and participating health care providers

|  | **Total population** | | **Operative** | | **Nonoperative** | |
| --- | --- | --- | --- | --- | --- | --- |
|  | n* |  | n* |  | n* |  |
| **Location of SDM^#^** | 66 |  | 24 |  | 42 |  |
| Emergency department |  | 30 (46%) |  | 5 (21%) |  | 25 (60%) |
| Ward |  | 50 (76%) |  | 22 (92%) |  | 28 (67%) |
| Preoperative holding |  | 2 (3%) |  | 0 |  | 2 (5%) |
| **Specialties participating in SDM^#^** | 77 |  | 27 |  | 50 |  |
| Orthopedic- or trauma surgery |  | 33 (43%) |  | 15 (56%) |  | 18 (36%) |
| (Orthopedic-) Surgery resident |  | 29 (38%) |  | 9 (33%) |  | 20 (40%) |
| Geriatrician |  | 42 (55%) |  | 18 (66%) |  | 24 (48%) |
| Emergency physician |  | 2 (3%) |  | 0 |  | 2 (4%) |
| Physician assistant GTU/trauma surgery |  | 18 (23%) |  | 4 (15%) |  | 14 (28%) |
| Others^+^ |  | 7 (9%) |  | 0 |  | 7 (14%) |

*Data are shown as median (P_25_-P_75_) or as n (%). n* refers to the number of patients for whom data were available.

^#^Participants can have had (part of the) SDM process at various locations with multiple healthcare providers from different specialties.

GTU, geriatric trauma unit; SDM, shared decision making

^+^Other specialties includes pulmonary medicine, cardiology, and/or intensive care medicine.
